# Evodiamine attenuates hyperuricemia-associated gouty arthritis and renal injury through urate-inflammatory axis modulation

**DOI:** 10.3389/fimmu.2026.1932250

**Published:** 2026-10-22

**Authors:** Yu Mou, Wenhao Liao, Qun Niu, Xue Zhan, Tianxin Zhang, YuChen Li, Hong Che, Peijia Liu, Xiangdong Yang, Jianyuan Tang, Zhilei Wang

**Affiliations:** 1 Department of Hematology, Hospital of Chengdu University of Traditional Chinese Medicine, Chengdu, China; 2 Department of Nephrology, the Key Laboratory for the Prevention and Treatment of Chronic Kidney Disease of Chongqing, Chongqing Clinical Research Center of Kidney and Urology Diseases, Xinqiao Hospital, Army Medical University (Third Military Medical University), Chongqing, China; 3 Department of Laboratory Medicine, the Third Affiliated Hospital of Zhengzhou University, Zhengzhou Key Laboratory for In Vitro Diagnosis of Hypertensive Disorders of Pregnancy, Zhengzhou, China; 4 Department of Laboratory Medicine, Hospital of Chengdu University of Traditional Chinese Medicine, Chengdu, China; 5 Sichuan Academy of Chinese Medicine Sciences, Chengdu, China; 6 TCM Prevention and Treatment of Metabolic and Chronic Diseases Key Laboratory of Sichuan Province, Hospital of Chengdu University of Traditional Chinese Medicine, Chengdu, China

**Keywords:** evodiamine, gouty arthritis, hyperuricemia, hyperuricemic nephropathy, inflammation, NLRP3 inflammasome, urate transporters

## Abstract

**Background:**

Therapeutic options for hyperuricemia (HUA) and its complications remain inadequate, particularly when gouty arthritis (GA) and hyperuricemic nephropathy (HN) coexist. Agents with combined urate-lowering, anti-inflammatory, and renoprotective effects are therefore needed. Evodiamine (EVO), a major alkaloid from *Evodia rutaecarpa*, exhibits anti-inflammatory and analgesic activities, but its role in HUA-related disorders has not been fully defined.

**Study design and methods:**

We examined the effects of *Evodia rutaecarpa* water extract (ERWE) and EVO in experimental models of HUA, HN, and GA.

**Results:**

In the HUA model, ERWE reduced plasma UA levels and increased urinary UA concentration, accompanied by reductions in ALT, AST, and selected inflammatory measurements. Metabolomic profiling indicated that these effects were associated with alterations in pyrimidine metabolism, α-linolenic acid metabolism, and butyrate-related pathways. In the HN model, EVO lowered serum UA and creatinine levels, improved renal pathology, and reduced renal URAT1 and GLUT9 expression. EVO also suppressed NLRP3/caspase-1/IL-1β signaling and reduced the expression of fibrosis-related markers, including α-SMA, COL1A1, and TIMP1. In the GA model, EVO alleviated monosodium urate (MSU)-induced ankle swelling, leukocyte infiltration, and inflammatory mediator production, including IL-1β, TNF-α, and MPO, while inhibiting NLRP3 inflammasome activation.

**Conclusion:**

ERWE and the alkaloid EVO showed significant protective effects in experimental HUA-related disorders. EVO, in particular, exerted urate-lowering, renoprotective, and anti-inflammatory activities, possibly through regulation of urate transporters and NLRP3 inflammasome signaling.

## Graphical Abstract

**Figure:**
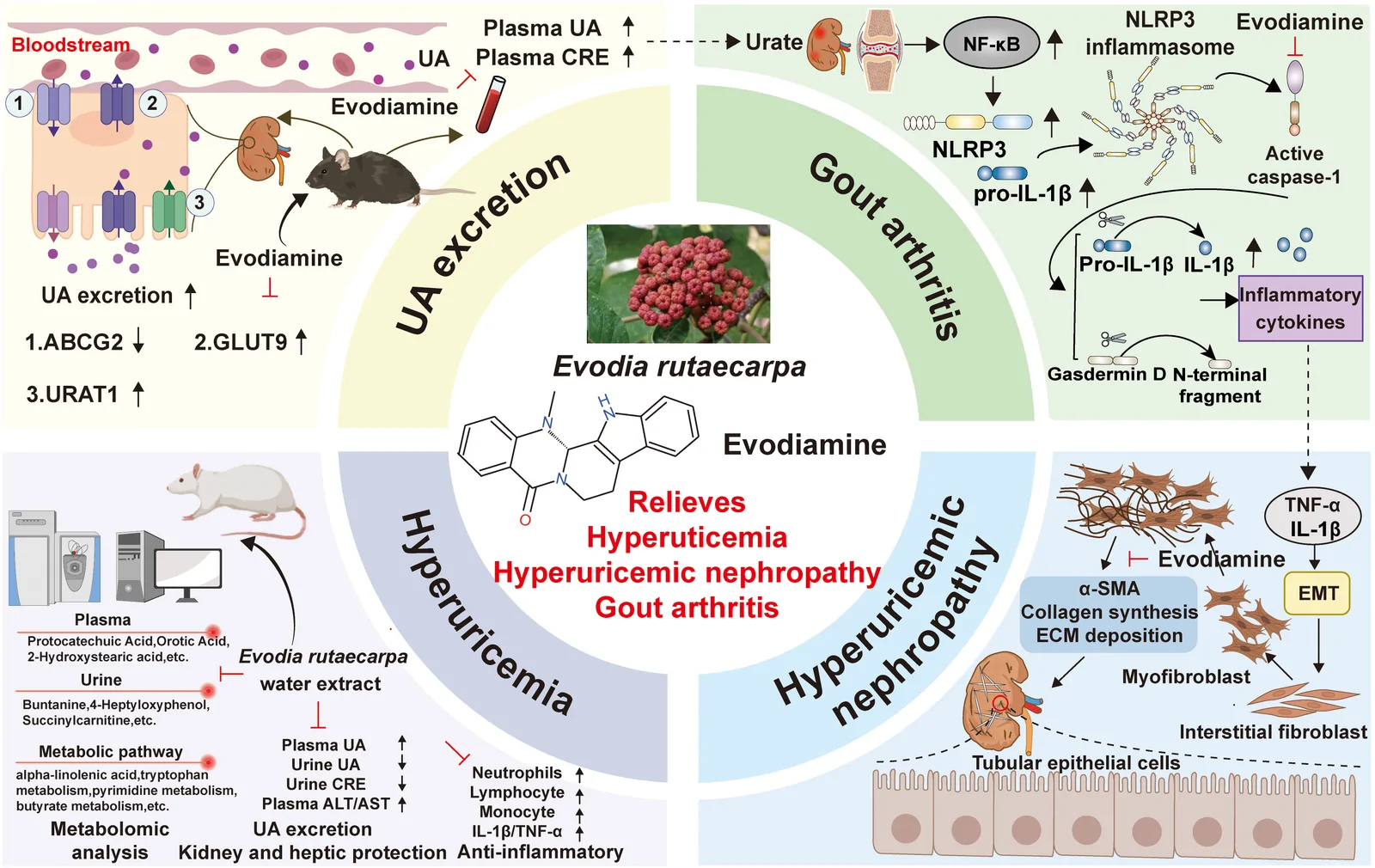

## Introduction

Hyperuricemia (HUA) is a common metabolic disorder caused by dysregulation of purine metabolism and is clinically defined by persistently elevated serum uric acid (UA) levels above the diagnostic thresholds of > 420 μmol/L in males and > 360 μmol/L in females (1, 2). As a key biochemical indicator for disease staging and therapeutic intervention, elevated UA is not only a hallmark of HUA but also a major pathogenic driver of gouty arthritis (GA), hyperuricemic nephropathy (HN), and other urate-associated comorbidities. Accordingly, a deeper understanding of the molecular mechanisms underlying HUA and the development of effective targeted therapies are of considerable clinical importance. In recent years, changes in dietary habits and lifestyle patterns have contributed to a steady increase in the prevalence of HUA worldwide, making it an increasingly important public health concern. In China, the prevalence of HUA in adults increased from 11.2% in 2015 to 14.0% in 2019, while the prevalence of GA rose concurrently from 1.1% to 1.6% over the same period (2, 3). These epidemiological trends highlight the urgent need for improved preventive strategies, earlier intervention, and more effective therapeutic approaches to reduce the growing burden of urate-related diseases on healthcare systems. Persistent HUA not only directly triggers acute gout attacks but also serves as an important modifiable risk factor for chronic kidney disease, cardiovascular disease, diabetes, and other metabolic disorders (4, 5). Taken together, these findings position HUA as a central therapeutic target in the management of systemic metabolic and inflammatory disorders.

The maintenance of serum UA homeostasis depends largely on tightly regulated renal urate handling. In proximal tubular epithelial cells, a coordinated network of urate transporters governs urate reabsorption and secretion. Among them, URAT1 and GLUT9 are primarily responsible for urate reabsorption, whereas OAT1 and ABCG2 facilitate urate excretion (6). Disruption of this transporter network contributes directly to hyperuricemia and its associated complications. When serum UA exceeds its solubility limit, MSU crystals form and deposit in joints, soft tissues, and renal parenchyma. These crystals are recognized by innate immune cells, leading to activation of toll-like receptors (TLRs) and the NLRP3 inflammasome. This cascade promotes caspase-1-mediated maturation and release of pro-inflammatory cytokines, including IL-1β, IL-6, and TNF-α, while also enhancing adhesion molecule expression (ICAM-1/VCAM-1), complement activation, and neutrophil recruitment. The resulting inflammatory amplification loop ultimately gives rise to the classical manifestations of acute gout, such as erythema, swelling, heat, and severe pain (7, 8). In parallel, chronic HUA induces renal microinflammation, oxidative stress, tubular epithelial injury, and progressive interstitial fibrosis, thereby contributing to the development of HN. Hallmark pathological changes of HN include glomerulosclerosis, tubular atrophy, and interstitial collagen deposition, reflecting sustained urate-mediated renal injury (9–12). In addition, MSU crystal deposition can further aggravate renal fibrosis by activating the NLRP3 inflammasome and promoting IL-1β secretion (13). These observations underscore the importance of controlling HUA not only to prevent acute gout flares but also to limit long-term renal damage.

Given the complex pathophysiology of HUA and its complications, the development of novel therapeutic agents with combined urate-lowering, renoprotective, and anti-inflammatory properties, together with acceptable safety profiles, has become a major research priority. Traditional Chinese medicine (TCM), characterized by its multi-component, multi-target, and holistic mode of action, offers unique advantages for the treatment of complex metabolic disorders. *Evodia rutaecarpa* (ER, Wu Zhu Yu), the dried nearly ripe fruit of *Euodia rutaecarpa* (Juss.) Benth., has been used in TCM for centuries and is recorded in classic medical texts such as *Sheng Nong’s Herbal Classic* as well as in the 2020 edition of the Chinese Pharmacopoeia (14). Modern pharmacological studies have demonstrated that ER and its bioactive constituents exert analgesic, anti-inflammatory, anti-atherosclerotic, antithrombotic, and metabolic regulatory effects, including modulation of hyperglycemia and hyperlipidemia (15–19). Historically, ER was also the principal ingredient of the traditional formula *Ji-Ming-Shan*, which was documented as having potent anti-gout and urate-lowering effects as early as the Southern Song Dynasty (20).

Evodiamine (EVO), an alkaloid constituent of ER, has attracted interest because of its reported pharmacological activities. Previous animal studies demonstrated that EVO reduced serum UA levels, partly through inhibition of xanthine oxidase, adenosine deaminase, and guanine deaminase (21, 22). In addition, EVO has been reported to modulate inflammatory responses, including NLRP3 inflammasome-related signaling, providing a pharmacological basis for its further investigation in HUA-related renal and gouty inflammation (23). EVO-derived formulations, such as butyryl-evodiamine-loaded lipid nanoparticles, have demonstrated therapeutic potential against HUA and its complications without causing obvious off-target organ toxicity (24). Nevertheless, the overall mechanisms by which EVO regulates urate homeostasis and attenuates HUA-associated inflammation, fibrosis, and acute gouty arthritis remain insufficiently understood. In the present study, we employed three *in vivo* models of HUA, HN, and GA to systematically investigate the therapeutic effects of Evodia rutaecarpa water extract (ERWE) and EVO. We examined renal URAT1 and GLUT9 expression at the mRNA and protein levels, *Abcg2* mRNA expression, and NLRP3 inflammasome-related signaling. By integrating pharmacodynamic evaluation across multiple disease models with mechanistic investigation at the molecular level, this study provides a stronger scientific basis for the further development and potential clinical application of EVO in HUA-related disorders.

## Materials and methods

### Animals

SPF-grade Sprague-Dawley (SD) rats and C57BL/6 mice (6–8 weeks old) were purchased from Chengdu Pharmaceutical Biotechnology Co., Ltd. Animals were housed in the Laboratory Animal Center of Chengdu University of Traditional Chinese Medicine under controlled conditions: temperature 22-25 °C, relative humidity 65-70%, 12 h light/12 h dark cycle, with free access to food and water. Prior to experiments, animals underwent a 7-day acclimatization period. All procedures were approved by the Animal Ethics Committee of Chengdu University of Traditional Chinese Medicine (Approval No. 2024024).

Animal species were selected based on the objectives and established use of each experimental model. SD rats were used for the short-term PO-induced HUA experiment, while C57BL/6 mice were used for the PO and adenine-induced HN and MSU-induced GA experiments. The three models were analyzed independently and were intended to provide complementary evidence across HUA-related pathological conditions; therefore, no direct interspecies comparison was performed.

### Preparation of ERWE

The dried slices of *Euodia rutaecarpa* (Batch No. 231001) were supplied by Sichuan Jinlin Pharmaceutical Co., Ltd. (Emei Mountain, China). The voucher specimen is deposited at the Sichuan Key Laboratory of Traditional Chinese Medicine for the Regulation of Metabolic Diseases. For the extraction protocol (25), 30 g of dried *Euodia rutaecarpa* was immersed in 200 mL of distilled water and allowed to soak for 30 min. The mixture was subsequently heated to 100 °C for 1 hour of aqueous extraction. Following extraction, the solution was centrifuged at 3500 rpm for 5 min to collect the supernatant. This supernatant was concentrated using a rotary evaporator and then subjected to freeze-drying to obtain the freeze-dried powder. The resulting ERWE freeze-dried powder had a total mass of 4.8 g, corresponding to a yield of 16% (w/w). The ER material used in the present study (Batch No. 231001) was the same botanical batch used in our previous study and was prepared using the identical aqueous extraction procedure. In that study, UPLC-QTOF-MS profiling of this batch-specific extract identified seven major chromatographic constituents, including dehydroevodiamine, evodine, evodol, evodiamine, rutaecarpine, evocarpine, and dihydroevocarpine (25). This previously reported chemical profile provides batch-specific chemical characterization of the ERWE used in the present study. The freeze-dried ERE powder was stored at −20 °C for subsequent experimental use.

### Active compounds of *Evodia rutaecarpa*

Evodiamine (T2868, TargetMol), an alkaloid constituent of ER, was used in this study. It has a molecular weight of 303.36, molecular formula C_19_H_17_N_3_O, CAS number 518-17-2, and a purity of 99.94% (batch number 114723).

### Construction of PO-induced HUA rat model and ERWE intervention

The HUA rat model was established via oral gavage of PO. SD rats were randomly assigned to four experimental groups: control (vehicle, n=8), model (model, n=8), low-dose ERWE (ERWE-L, 0.263 g ER/kg body weight, n=8), and high-dose ERWE (ERWE-H, 0.525 g ER/kg body weight, n=8). The ERWE doses were expressed as raw-herb equivalents and represented the low- and high-dose levels evaluated in the present study. Previous pharmacological evidence supporting the uric acid-lowering and anti-inflammatory effects of Euodiae Fructus extract has been reported in our earlier study (25). All groups received standard chow and water ad libitum. Control rats were administered carboxymethylcellulose sodium (CMC-Na) as vehicle, while both model and ERWE-treated groups received PO (250 mg/kg) via gavage. One hour post-PO administration, ERWE-L and ERWE-H groups were gavaged with their respective doses. This treatment regimen was repeated daily for three consecutive days. Two hours after the final administration of ERWE, orbital venous blood samples were collected. Immediately following collection, whole blood samples were analyzed for lymphocyte, neutrophil, and monocyte counts using a fully automated hematology analyzer. The remaining blood was processed to isolate serum through centrifugation at 1300 g for 10 minutes at 4 °C. The separated plasma was aliquoted for subsequent biochemical analyses, while the residual plasma was utilized for the measurement of UA, creatinine (CRE), and blood urea nitrogen (BUN) using standard laboratory protocols. Urine was collected over a 24-h period using metabolic cages, and urinary UA and creatinine were measured using standard biochemical assays, and 24-h urinary UA excretion was calculated based on urinary UA concentration and the corresponding 24-h urine volume. Moreover, untargeted metabolomics was employed to analyze metabolic changes in urine and plasma samples, providing comprehensive profiling of endogenous metabolites and pathway alterations.

### Construction of PO/adenine-induced HN mouse model and EVO intervention

The HN model was induced via combined administration of PO and adenine in C57BL/6 mice (26–28). After a 1-week acclimatization period, fifty mice were randomly allocated into five experimental groups (n=10/group): control (Ctrl), model (PO+Adenine), benzbromarone (20 mg/kg), low-dose EVO (EVO-L, 20 mg/kg), and high-dose EVO (EVO-H, 40 mg/kg). The EVO doses of 20 and 40 mg/kg were selected with reference to previously reported oral dose ranges in murine pharmacological studies (29). All groups received standard chow and water ad libitum. Control mice were administered CMC-Na via oral gavage. The model and treatment groups received PO (100 mg/kg) combined with adenine (37.5 mg/kg) once daily for 4 weeks. From day 14 onwards, while maintaining PO+adenine administration, the benzbromarone, EVO-L, and EVO-H groups received their respective pharmacological interventions daily for 21 consecutive days. The control group continued to receive CMC-Na throughout the intervention period. Post-final administration, plasma and renal tissues were harvested from mice. A portion of plasma was allocated for immediate biochemical analysis of serum UA and CRE. The remaining plasma was aliquoted and cryopreserved at −80 °C for subsequent ELISA assays. For renal tissues, the left kidney was rinsed with PBS, blotted dry, and weighed before immediate fixation in 4% paraformaldehyde for histological evaluation. The right kidney was snap-frozen in liquid nitrogen within 30 seconds of harvesting and stored at −80 °C for molecular analyses, including Western blot protein quantification and quantitative real-time PCR (qPCR) gene expression profiling.

### Construction of MSU-induced GA mouse model and EVO intervention

Mice models of GA were used as described previously (30). After a 1-week acclimatization period, thirty C57BL/6 mice were randomly allocated into five experimental groups (n=6/group): control (Ctrl), model (monosodium urate crystal, MSU), colchicine (1 mg/kg), low-dose EVO (EVO-L, 20 mg/kg), and high-dose EVO (EVO-H, 40 mg/kg). All groups received daily oral gavage administration for 7 consecutive days. One hour post-final administration, acute GA was induced in all groups except the control via intra-articular injection of 50 μL MSU crystals (25 mg/ml) (U2875, Sigma-Aldrich, USA) into the posterior aspect of the left ankle joint. Paw swelling was measured using digital calipers at 0, 3, 6, 12, 24, and 48 hours post-MSU injection to quantify inflammatory response. Post-48-hour MSU injection, plasma and ankle joint tissues were harvested from mice. A portion of plasma was allocated for immediate biochemical analysis of serum UA and myeloperoxidase (MPO) concentrations. The remaining plasma was aliquoted and cryopreserved at −80 °C for subsequent ELISA assays. For ankle joint tissues, a subset was fixed in 4% paraformaldehyde for histological examination and immunohistochemical detection of IL-1β. The remaining ankle joints were snap-frozen in liquid nitrogen and stored at −80 °C for subsequent Western blot protein analysis.

### Blood routine and biochemical indicators

Centrifuged plasma samples were analyzed using a fully automated chemistry analyzer (BS-240VET, Shenzhen Myriad Biomedical Electronics Co., Ltd.) to quantify complete blood count parameters including total white blood cells, neutrophils, monocytes, and lymphocytes, as well as biochemical parameters comprising UA (E-BC-K016-M, Elabscience), CRE (E-BC-K188-M, Elabscience), BUN (E-BC-K016-M, Elabscience), and serum alanine aminotransferase (ALT) and aspartate aminotransferase (AST).

### ELISA

Inflammatory cytokine levels were quantified using mouse-specific ELISA kits: TNF-α (E-EL-M3063, Elabscience), IL-1β (E-EL-M0037, Elabscience), and IL-6 (JM-02446M2, Jingmei Biotechnology) following the manufacturers’ protocols strictly.

### MPO measurement

MPO levels were determined using a MPO colorimetric assay kit (E-BC-K074, Elabscience) strictly following the manufacturer’s protocols.

### Histopathology and immunohistochemistry

Renal tissues from mice underwent fixation, embedding, sectioning, followed by H&E, Masson staining, and periodic acid-Schiff (PAS) staining. Optical microscopy was employed to examine renal tissue sections for pathological changes including inflammation, damage, and fibrosis in renal tubules, glomeruli, and interstitial regions. Ankle joint tissues were similarly processed through fixation, embedding, and sectioning, then subjected to H&E staining to evaluate synovial inflammation, and immunohistochemical staining to detect IL-1β (16806-1-AP, Proteintech) expression using antibody.

### Real-time PCR analysis

Total RNA was isolated from homogenized tissues using the RNA Easy Fast Tissue/Cell Kit (DP451, TIANGEN) following the manufacturer’s protocols. Complementary DNA (cDNA) was synthesized using the iScript cDNA Synthesis Kit (1708891, Bio-Rad) as per the manufacturer’s instructions. Quantitative PCR was performed with the QuantiNova SYBR Green PCR Kit (208054, TIANGEN) according to the provided guidelines. Primer sequences for the target genes are listed in **Table 1**.

**Table 1:** Primer sequences for RT-qPCR analysis.

Genes	Forward primers (5′-3′)	Reverse primers (5′-3′)
*Slc2a9*	TTGCTTTAGCTTCCCTGATGTG	GAGAGGTTGTACCCGTAGAGG
*Slc22a12*	CGTGGGACCTGGTATGTAACT	CCAAACCTATCTGAGGCATGG
*Abcg2*	CTAGGGGCCGAGGCTTATAC	AGTGTTGCTACAGACACCACA
*Il1b*	GAAATGCCACCTTTTGACAGTG	CTGGATGCTCTCATCAGGACA
*Nlrp3*	ATTACCCGCCCGAGAAAGG	TCGCAGCAAAGATCCACACAG
*Casp1*	ACAAGGCACGGGACCTATG	TCCCAGTCAGTCCTGGAAATG
*Pycard*	CTTGTCAGGGGATGAACTCAAAA	GCCATACGACTCCAGATAGTAGC
*Col1a1*	GCTCCTCTTAGGGGCCACT	CCACGTCTCACCATTGGGG
*Acta2*	GTCCCAGACATCAGGGAGTAA	TCGGATACTTCAGCGTCAGGA
*Ctgf*	GGGCCTCTTCTGCGATTTC	ATCCAGGCAAGTGCATTGGTA
*Ldlr*	CTGTGGGCTCCATAGGCTATCT	GCGGTCCAGGGTCATCTTC
*Actb*	GTGACGTTGACATCCGTAAAGA	GCCGGACTCATCGTACTCC
*Gapdh*	GTCATCCCAGAGCTGAACG	TCATACTTGGCAGGTTTCTCC

### Western blot

Total proteins were extracted from homogenized tissues using RIPA lysis buffer containing protease inhibitors. Protein concentration was quantified using a BCA protein assay kit (Beyotime, P0010). Equal amounts of protein (30 μg) were separated by SDS-PAGE and transferred onto PVDF membranes (Millipore, IEVH00005). Membranes were blocked with 5% non-fat milk in TBST for 1 h at room temperature, followed by overnight incubation at 4 °C with primary antibodies. After washing, membranes were incubated with HRP-conjugated secondary antibodies for 1 h. Protein bands were visualized using enhanced chemiluminescence (ECL) substrate (Oriscience, PD203) and imaged with a GelView 6000Pro SE imaging system.

### Antibodies

Anti-rabbit-GLUT9 (26486-1-AP, Proteintech), anti-rabbit-URAT1 (14937-1-AP, Proteintech), anti-mouse-NLRP3 (AG-20B-0014, AdipoGen), anti-mouse-Caspase-1 (AG-20B-0042, AdipoGen), anti-rabbit-Asc (67824, Cell Signaling Technology), anti-mouse cleaved-IL-1β (AF-401-NA, R&D Systems), anti-rabbit-LDLR (10785-1-AP, Proteintech), anti-rabbit-TIMP1 (16644-1-AP, Proteintech), anti-rabbit-α-SMA (14395-1-AP, Proteintech), anti-GAPDH (60,004-1–1 g, Proteintech), anti-Lamin B1 (12,987-1-AP, Proteintech), anti-β-actin (81,115-1-RR, Proteintech), goat anti-Mouse IgG (SA00001-1, Proteintech), and goat anti-Rabbit IgG (SA00001-2, Proteintech).

### Untargeted metabolomics analysis

Plasma and urine samples (100 μL; n = 3 animals/group) were extracted with 400 μL acetonitrile/methanol (1:1) containing 0.02 mg/mL L-2-chlorophenylalanine. After vortexing (30 s), sonication (5 °C, 40 kHz, 30 min), incubation (−20 °C, 30 min), and centrifugation (13,000 × g, 4 °C, 15 min), supernatants were nitrogen-dried and reconstituted in 100 μL acetonitrile/water (1:1). Following sonication (5 min) and centrifugation (10 min) under the same conditions, samples were analyzed using an HSS T3 column (100 × 2.1 mm, 1.8 μm) coupled to a Q Exactive HF-X in positive and negative electrospray modes. Pooled QC samples were injected every 5–15 samples.

Progenesis QI was used for peak processing and putative metabolite annotation against HMDB, METLIN, and the Majorbio database. Features detected in ≥80% of samples underwent minimum-value imputation, total-signal normalization, QC filtering (RSD ≤30%), and log10 transformation. PCA and OPLS-DA used ropls (v1.6.2), with seven-fold cross-validation for OPLS-DA. Potentially differential metabolites met VIP >1 and Student’s t-test P <0.05 and were interpreted as exploratory.

### Statistical analysis

Statistical analyses of basic experiments were conducted using Prism 9 (GraphPad Software). All datasets are presented as mean ± SEM. Comparative analyses employed unpaired Student’s t-test for two-group comparisons or one-way ANOVA with Dunnett’s *post hoc* test for multi-group comparisons. Significance threshold was defined as *P* < 0.05.

## Results

### The regulatory effects of ERWE on UA production and excretion, hepatorenal protection, and anti-inflammatory processes in HUA rat models

To evaluate the pharmacological effects of ERWE on hyperuricemia, a PO-induced HUA rat model was established (**Figure 1**). Compared with the control group, PO administration significantly increased plasma UA levels, supporting successful establishment of the hyperuricemic phenotype in SD rats, whereas ERWE treatment significantly reduced plasma UA levels in a dose-dependent manner (**Figure 1**). Plasma creatinine and blood urea nitrogen levels showed no significant differences among the groups (**Figure 1C**), indicating that ERWE did not adversely affect renal function-related biochemical indices under the present experimental conditions. 24-h urinary creatinine and UA excretion were decreased in PO-induced HUA rats compared with the control group, whereas ERWE treatment partially restored these changes in a dose-dependent manner (**Figure 1E**). In particular, the restoration of 24-h urinary UA excretion suggests that ERWE may improve impaired urinary urate elimination in HUA rats. In addition, PO-induced HUA significantly increased plasma AST and ALT levels, whereas ERWE administration attenuated these elevations in a dose-dependent manner (**Figure 1G**), indicating an improvement in HUA-associated hepatic injury. Hematological analysis further showed that circulating monocytes, lymphocytes, and neutrophils were increased in the model group compared with the control group, and these alterations were significantly reduced by ERWE treatment (**Figure 1I**). Consistently, the elevated plasma levels of IL-1β and TNF-α in HUA rats were also decreased after ERWE intervention (**Figure 1L**). Together, these results demonstrate that ERWE ameliorates PO-induced hyperuricemia by reducing circulating UA levels, improving hepatic dysfunction, and suppressing systemic inflammatory responses.

**Figure 1:**
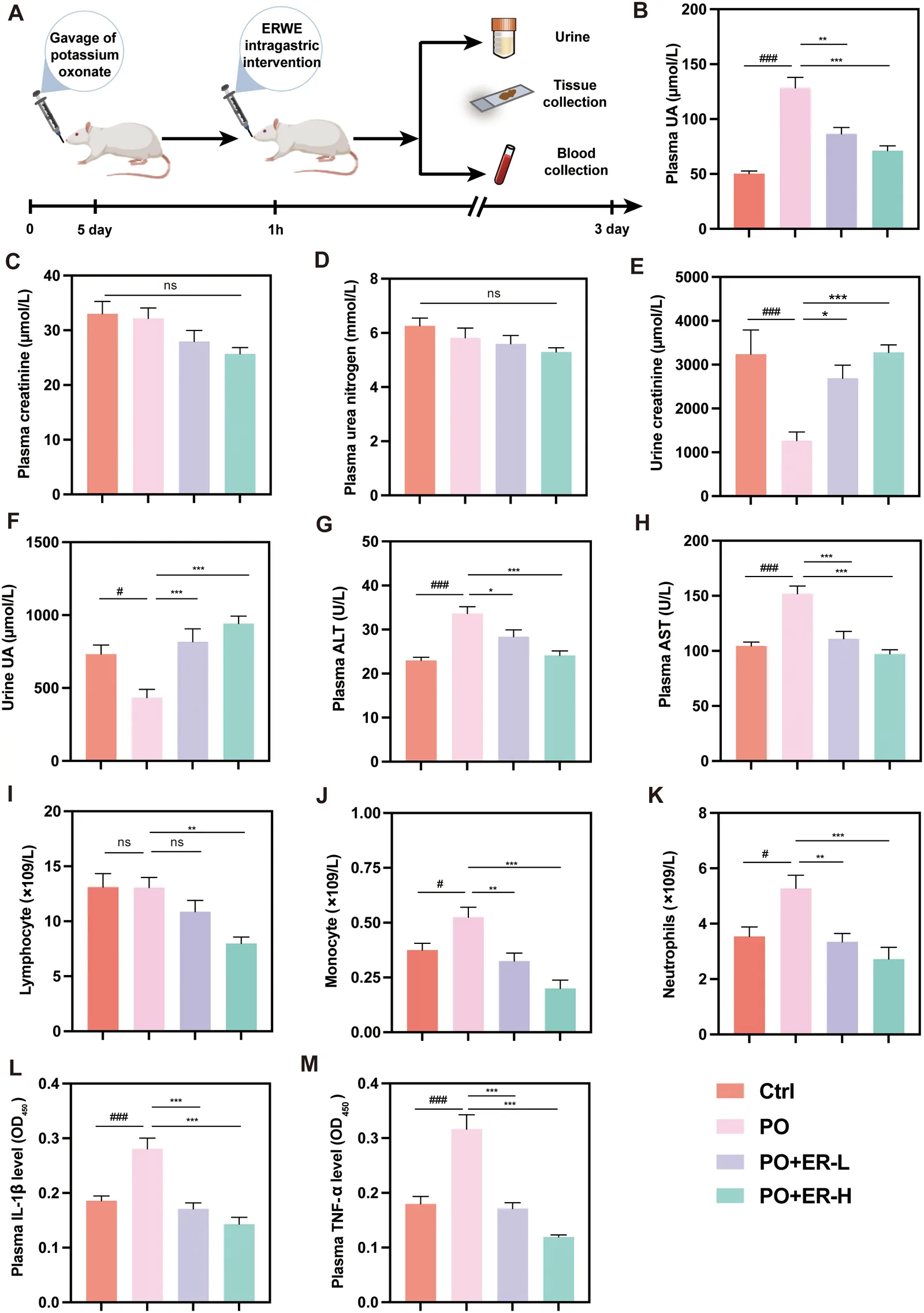
ERWE treatment ameliorates HUA and associated hepatorenal and inflammatory dysfunction. **(A)** Experimental design. **(B–D)** Plasma UA, BUN and creatinine in rat of different groups. **(E, F)** 24 h urinary creatinine and UA excretion in rats from different groups. **(G, H)** Plasma ALT and AST in rat of different groups. **(I–K)** The level of lymphocytes, monocytes, and neutrophils. **(L, M)** ELISA analysis of plasma IL-1β and TNF-α levels in rats. Data are expressed as mean ± SEM. ns, not significant. ^#^*P* < 0.05, ^###^*P* < 0.001 vs. the group of Ctrl and ^*^P < 0.05, ^**^P < 0.01, ^***^P < 0.001 vs. the group of PO.

### Regulatory effects of ERWE on metabolic characteristics in HUA rats

Untargeted metabolomics was performed to characterize metabolic changes associated with ERWE treatment in PO-induced HUA rats. Plasma and urine samples from three independent animals per group were analyzed using a UHPLC–Q Exactive HF-X platform in positive and negative electrospray ionization modes. Representative total ion chromatograms are shown in **Supplementary Figure S1**. PCA revealed partial separation between the Ctrl and Model groups (**Figure 2A**; **Supplementary Figure S2A**). PLS-DA and OPLS-DA plots displayed treatment-associated differences, with ERWE-treated samples showing shifts toward the Ctrl group in some comparisons (**Figure 2C**; **Supplementary Figure S2**).

**Figure 2:**
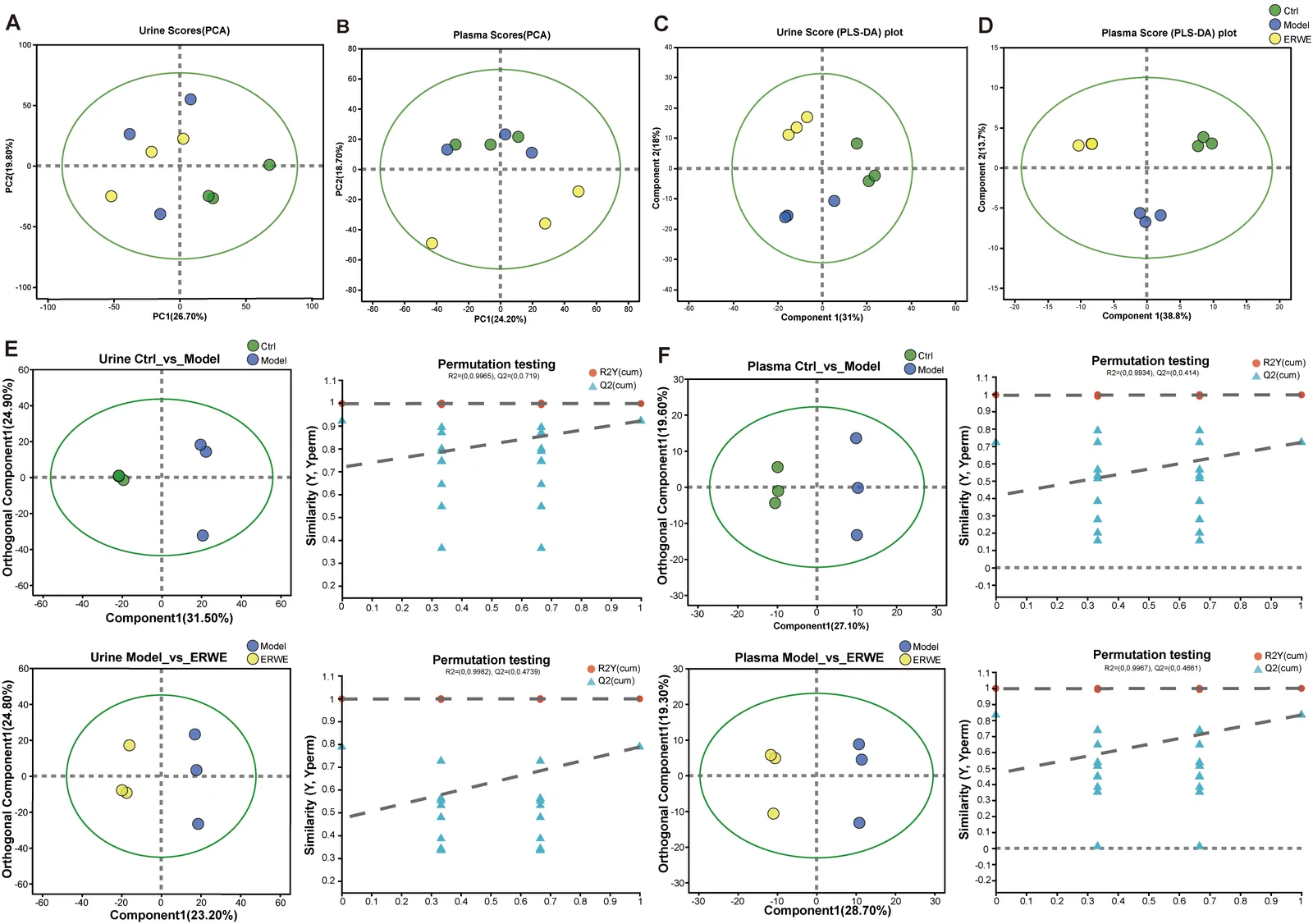
PCA and OPLS-DA analysis of urinary and plasma metabolites in rat cecal contents across different treatment groups. PCA scores plot of urinary **(A)** and plasma **(B)** metabolites between Ctrl, Model and ERWE group; OPLS-DA scores plot of urinary **(C)** and plasma **(D)** metabolites between Ctrl, Model and ERWE group; OPLS-DA analysis of urinary **(E)** and plasma **(F)** metabolites comparing Ctrl vs. Model and Model vs. ERWE.

Using VIP >1 and P <0.05 as screening criteria, overlapping comparisons of Ctrl versus Model and Model versus ERWE identified six candidate differential metabolites in plasma and 20 in urine (**Figure 3C**). Hierarchical clustering illustrated distinct abundance patterns among groups, with several metabolites changing toward control levels following ERWE treatment (**Figure 3A, B**). In plasma, putatively annotated protocatechuic acid and 2-hydroxystearic acid were decreased in the Model group and increased after ERWE treatment. Orotic acid and 3-hydroxybutanoic acid also exhibited group-related differences, suggesting alterations in nucleotide and energy metabolism (**Table 2**). Urinary candidates included succinylcarnitine, N-lactoylphenylalanine, and 5-methoxyindoleacetate, among other metabolites associated with organic acid and amino acid metabolism (**Table 3**).

**Figure 3:**
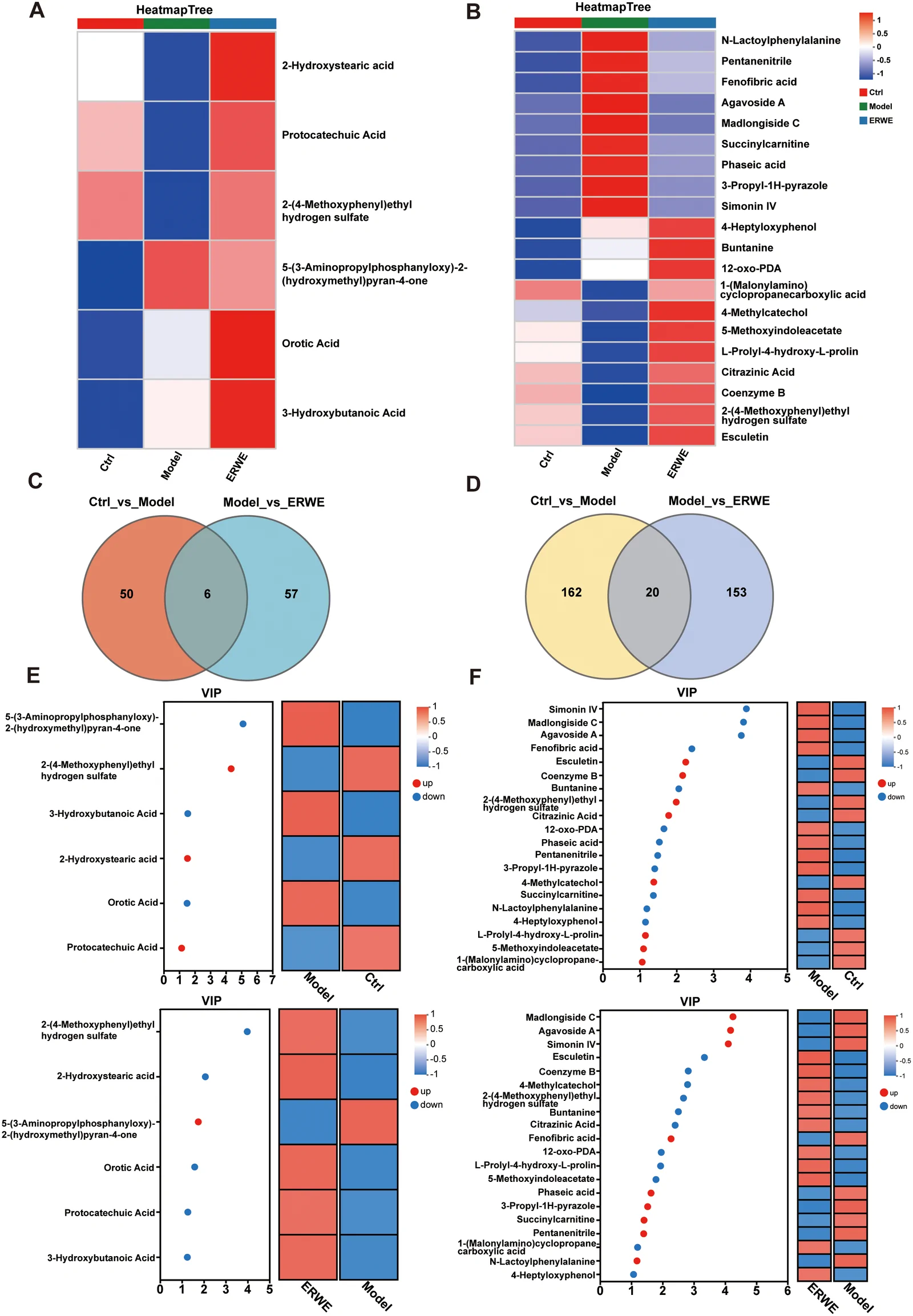
Metabolomic profiling of urinary metabolites in rats across different treatment groups. Heatmap of selected metabolites **(A)** and additional metabolites **(B)** in the Ctrl, Model, and ERWE groups; Venn diagrams comparing metabolites between Ctrl vs. Model **(C)** and Model vs. ERWE **(D)** groups; VIP scores plot **(E)** and corresponding metabolite rankings **(F)** distinguishing the Ctrl, Model, and ERWE groups. Data are presented as mean ± SEM. VIP analysis was used to identify key metabolites, and heatmaps and Venn diagrams were employed to display differences.

**Table 2:** Differences in plasma metabolism.

Metabolite name	Formula	Retention time (min)	m/z	Model_vs_Ctrl	ERWE_vs_Model
Protocatechuic Acid	C_7_H_6_O_4_	6.142	153.019	Down	Up
2-Hydroxystearic acid	C_18_H_36_O_3_	6.164	299.259	Down	Up
2-(4-Methoxyphenyl)ethyl hydrogen sulfate	C_9_H_12_O_5_S	5.683	231.033	Down	Up
Orotic Acid	C_5_H_4_N_2_O_4_	0.733	155.009	Up	Up
5-(3-Aminopropylphosphanyloxy)-2-(hydroxymethyl)pyran-4-one	C_9_H_13_NO_4_P	0.780	459.112	Up	Down
3-Hydroxybutanoic Acid	C_4_H_8_O_3_	2.076	103.039	Up	Up

**Table 3:** Differential metabolism of urine.

Metabolite name	Formula	Retention time (min)	m/z	Model_vs_Ctrl	ERWE_vs_Model
1-(Malonylamino)cyclopropanecarboxylic acid	C_7_H_9_NO_5_	1.746	188.055	Down	Up
N-Lactoylphenylalanine	C_12_H_15_NO_4_	2.332	238.108	Up	Down
Succinylcarnitine	C_11_H_19_NO_6_	1.690	262.129	Up	Down
3-Propyl-1H-pyrazole	C_6_H_10_N_2_	1.936	111.092	Up	Down
Buntanine	C_20_H_21_NO_5_	2.078	378.129	Up	Up
Coenzyme B	C_11_H_22_NO_7_PS	3.111	308.070	Down	Up
Agavoside A	C_33_H_52_O_9_	4.409	606.370	Up	Down
Madlongiside C	C_35_H_56_O_10_	4.497	650.396	Up	Down
4-Heptyloxyphenol	C_13_H_20_O_2_	5.812	191.143	Up	Up
5-Methoxyindoleacetate	C_11_H_11_NO_3_	5.565	188.071	Down	Up
12-oxo-PDA	C_18_H_28_O_3_	5.557	310.238	Up	Up
L-Prolyl-4-hydroxy-L-prolin	C_10_H_16_N_2_O_4_	4.704	193.097	Down	Up
Simonin IV	C_68_H_120_O_24_	4.527	672.409	Up	Down
Pentanenitrile	C_5_H_9_N	1.967	125.108	Up	Down
2-(4-Methoxyphenyl)ethyl hydrogen sulfate	C_9_H_12_O_5_S	5.700	231.033	Down	Up
Phaseic acid	C_15_H_20_O_5_	5.818	279.124	Up	Down
Fenofibric acid	C_17_H_15_ClO_4_	5.654	353.034	Up	Down
4-Methylcatechol	C_7_H_8_O_2_	4.788	123.044	Down	Up
Esculetin	C_9_H_6_O_4_	3.908	177.019	Down	Up
Citrazinic Acid	C_6_H_5_NO_4_	2.750	154.014	Down	Up

KEGG analysis highlighted α-linolenic acid, tryptophan, and methane metabolism among the pathways associated with urinary candidates, and pyrimidine metabolism, butyrate metabolism, and cofactor biosynthesis among those associated with plasma candidates (**Supplementary Figures S3A, B**). Together, these observations provide a preliminary metabolic characterization of ERWE intervention and identify candidate pathways that may be relevant to its pharmacological effects. Given the limited biological replication, the metabolite and pathway associations require confirmation in an independent cohort.

In complementary experiments, EVO, an alkaloid constituent of ER, was evaluated in the PO/adenine-induced HN model to investigate its effects on HUA-associated renal dysfunction, inflammation, and structural injury.

### EVO reduces serum UA levels and ameliorates renal dysfunction and pathological lesions in HN mice

A PO/adenine-induced HN mouse model was established to evaluate the urate-lowering and renoprotective effects of EVO (**Figure 4**). Compared with the control group, HN model mice exhibited significant body weight loss, whereas treatment with benzbromarone or EVO at 20 and 40 mg/kg significantly alleviated this reduction (**Figure 4**). Kidney weight was decreased in the model group, while no significant difference was observed in the renal index among the groups (**Figure 4C**). Serum biochemical analysis showed that PO/adenine administration markedly increased serum UA and creatinine levels, indicating hyperuricemia accompanied by renal dysfunction. EVO treatment reduced serum UA levels in a dose-dependent manner and significantly decreased serum creatinine levels, with the high-dose EVO group showing a more pronounced effect (**Figure 4E**). Consistently, histopathological examination revealed obvious renal tubular injury in HN mice, whereas EVO treatment attenuated these pathological lesions, as reflected by reduced tubular injury scores (**Figure 4G**). These results indicate that EVO effectively alleviates hyperuricemia-associated renal dysfunction and pathological damage in HN mice.

**Figure 4:**
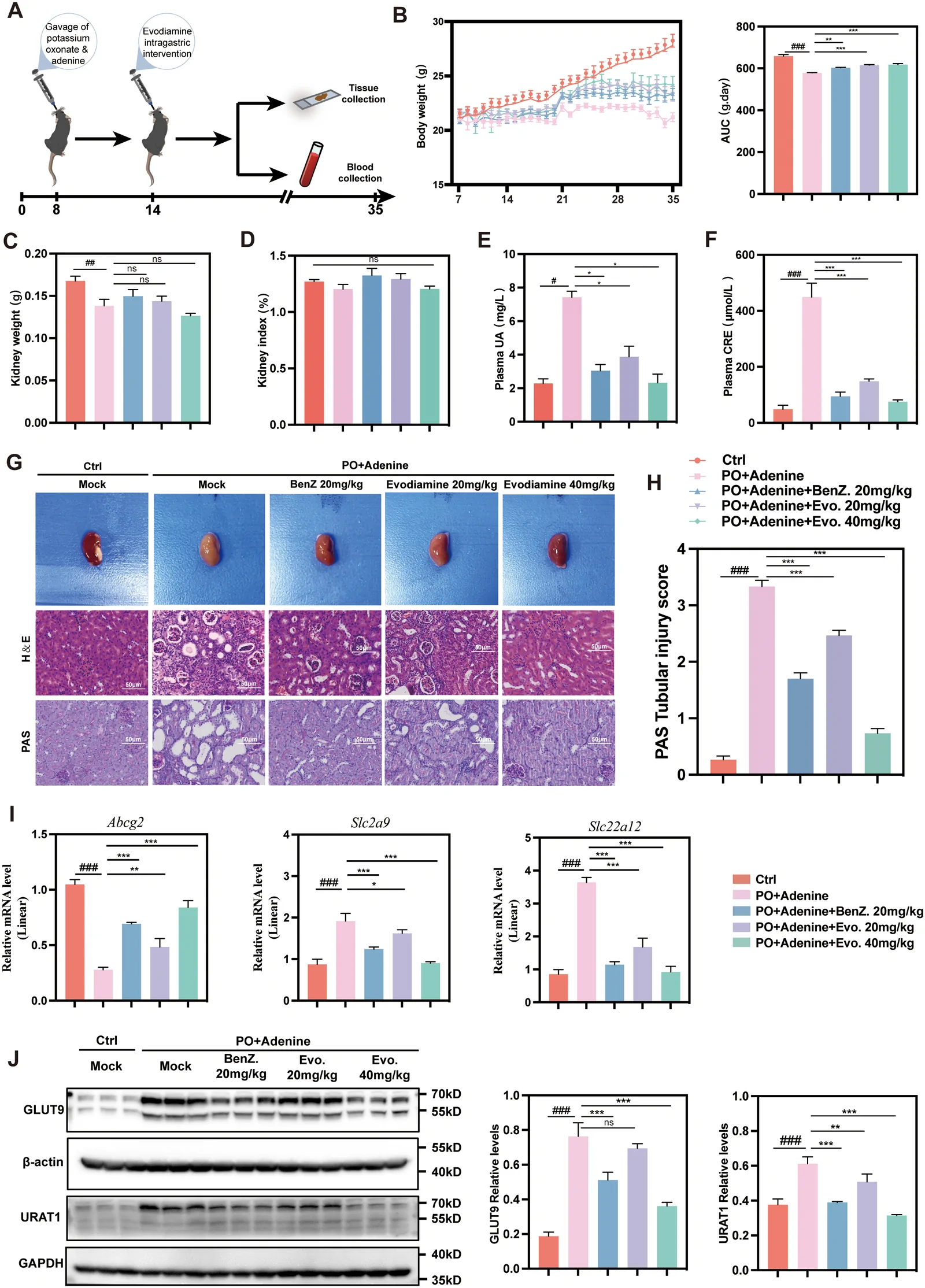
EVO improves renal function and pathological damage, and regulates the expression of UA transporters in HN mice. **(A)** Experimental design. **(B)** Body weight change over time and area under the curve (AUC) for body weight data. **(C, D)** Kidney weight **(C)** and kidney index (kidney weight/body weight ratio) **(D)** in different groups. **(E, F)** Plasma UA **(E)** and creatinine **(F)** levels. **(G)** H&E and PAS staining (Scale bar: 50 μm) in mice kidney sections. **(H)** PAS tubular injury score. **(I)** Relative expression of *Abcg2*, *Slc2a9*, *Slc22a12* mRNA in mice kidney. **(J)** Western blot analysis of URAT1, and GLUT9 in mice kidney. Relative intensity of URAT1, and GLUT9 were determined. Data are expressed as mean ± SEM from three independent experiments with biological duplicates. ns, not significant. ^#^*P* < 0.05, ^##^*P* < 0.01, ^###^*P* < 0.001 vs. the group of Ctrl and ^*^P < 0.05, ^**^P < 0.01, ^***^P < 0.001 vs. the group of PO + Adenine.

To further investigate whether the urate-lowering effect of EVO was associated with renal urate handling, the expression of key urate transporters was examined. RT-qPCR analysis showed that the mRNA levels of the urate reabsorption transporters *Slc22a12* (URAT1) and *Slc2a9* (GLUT9) were significantly increased in the kidneys of HN mice, whereas the expression of the urate efflux transporter *Abcg2* was decreased (**Figure 4**). EVO treatment reversed these abnormalities in a dose-dependent manner, particularly at 40 mg/kg. Consistent with the transcriptional results, Western blot analysis further confirmed that high-dose EVO reduced the protein expression of URAT1 and GLUT9 in renal tissues (**Figure 4**). These findings suggest that EVO may improve urate homeostasis by suppressing renal urate reabsorption and enhancing urate excretion, thereby contributing to its urate-lowering and renoprotective effects in HN mice.

### EVO ameliorates renal interstitial fibrosis in HN mouse models

Following the improvement in renal dysfunction and urate transporter dysregulation observed after EVO treatment, we further examined whether EVO alleviated renal interstitial fibrosis in HN mice. Compared with the control group, HN mice showed marked upregulation of fibrosis-related genes, including *Col1a1*, *Acta2*, *Ctgf*, and *Ldlr*, in renal tissues (**Figure 5**). Consistently, Western blot analysis showed increased protein expression of LDLR, α-SMA, and TIMP1 in the kidneys of HN mice (**Figure 5**). EVO treatment, particularly at 40 mg/kg, significantly reduced the mRNA levels of *Col1a1*, *Acta2*, *Ctgf*, and *Ldlr*, and decreased the protein expression of LDLR, α-SMA, and TIMP1 (**Figure 5A**). Histological analysis further supported these findings. H&E and Masson staining revealed obvious tubular injury, inflammatory cell infiltration, and collagen deposition in the kidneys of HN mice, whereas EVO treatment markedly attenuated these pathological alterations (**Figure 5**). Taken together, these results indicate that EVO mitigates renal interstitial fibrosis in HN mice, which may be associated with reduced extracellular matrix accumulation and suppression of fibrosis-related signaling.

**Figure 5:**
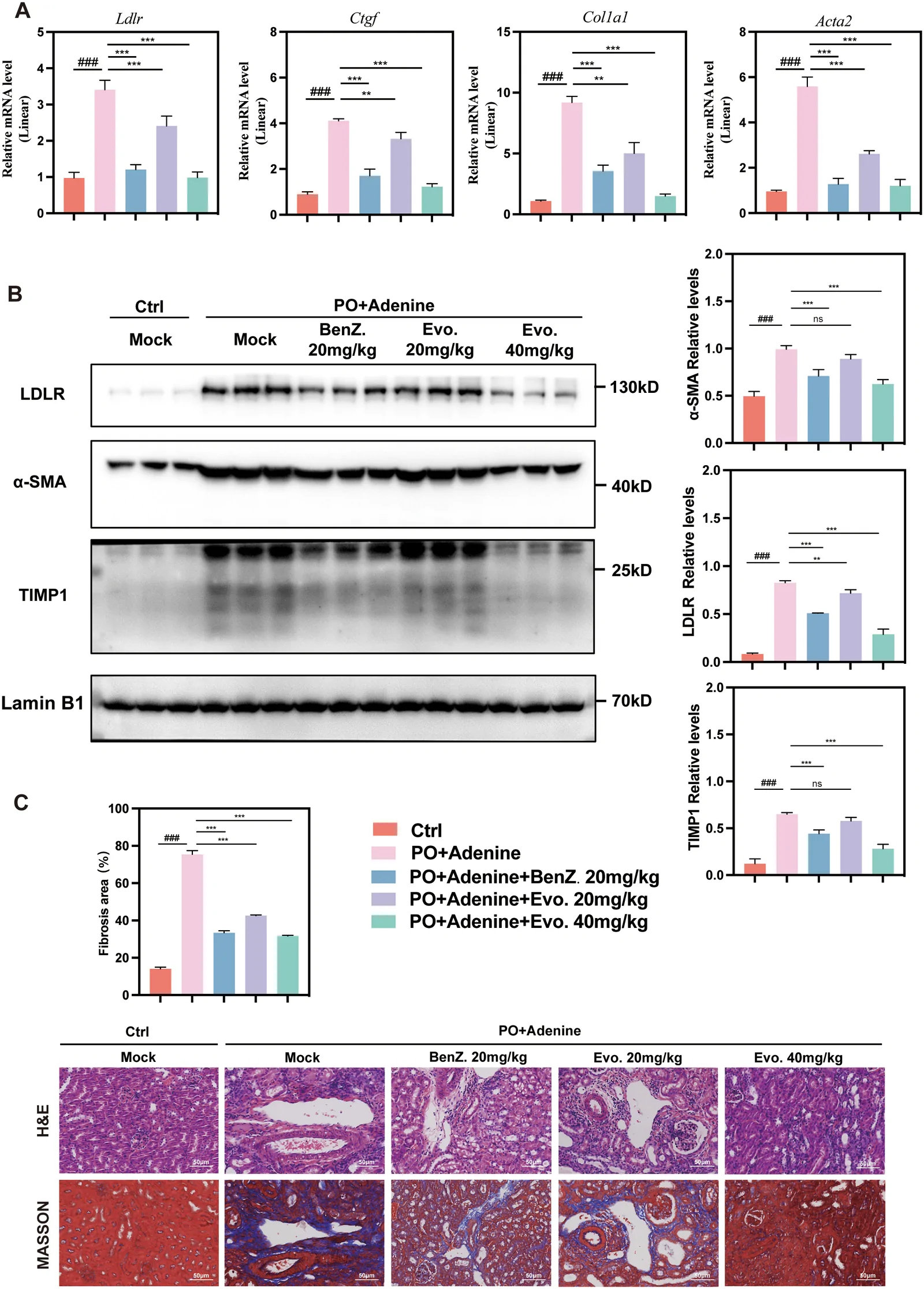
EVO ameliorates renal interstitial fibrosis. **(A)** Relative expression of *Ldlr, Ctgf, Acta2*, and *Col1a1* mRNA in mice kidney. **(B)** Western blot analysis of LDLR, α-SMA, and TIMP1 in mice kidney. Relative intensity of LDLR, α-SMA, and TIMP1 were determined. **(C)** Masson staining (Scale bar: 50 μm) in mice kidney sections. Relative intensity of collagen volume fraction were determined. Data are expressed as mean ± SEM from three independent experiments with biological duplicates. ns, not significant. ^###^*P* < 0.001 vs. the group of Ctrl and ^*^P < 0.05, ^**^P < 0.01, ^***^P < 0.001 vs. the group of PO + Adenine.

### EVO inhibits the activation of the NLRP3/caspase-1/IL-1β axis in HN mouse models

Given that EVO markedly attenuated renal interstitial fibrosis in HN mice, we next examined whether this effect was associated with the regulation of inflammatory responses. Compared with the control group, PO/adenine-induced HN mice showed significantly increased serum levels of TNF-α and IL-6, whereas serum IL-1β displayed an increasing trend but did not reach statistical significance (**Figure 6A**). Consistent with the systemic inflammatory changes, renal mRNA expression levels of *Nlrp3*, *Casp1*, and *Il1b* were markedly upregulated in the model group (**Figure 6**). Western blot analysis further showed increased protein expression of NLRP3, cleaved Caspase-1 p20, and mature IL-1β p17 in renal tissues, indicating activation of the NLRP3/Caspase-1/IL-1β inflammasome axis in HN mice (**Figure 6**). EVO treatment, particularly at 40 mg/kg, significantly reduced serum TNF-α and IL-6 levels and suppressed the renal expression of NLRP3 inflammasome-related genes and proteins (**Figures 6A, C**). Although the change in serum IL-1β was not statistically significant, EVO reduced renal *Il1b* expression and mature IL-1β p17 protein levels, suggesting that its anti-inflammatory effect was more evident at the renal tissue level. These findings indicate that EVO attenuates renal inflammatory activation in HN mice, which may contribute to its anti-fibrotic and renoprotective effects through suppression of the NLRP3/Caspase-1/IL-1β signaling pathway.

**Figure 6:**
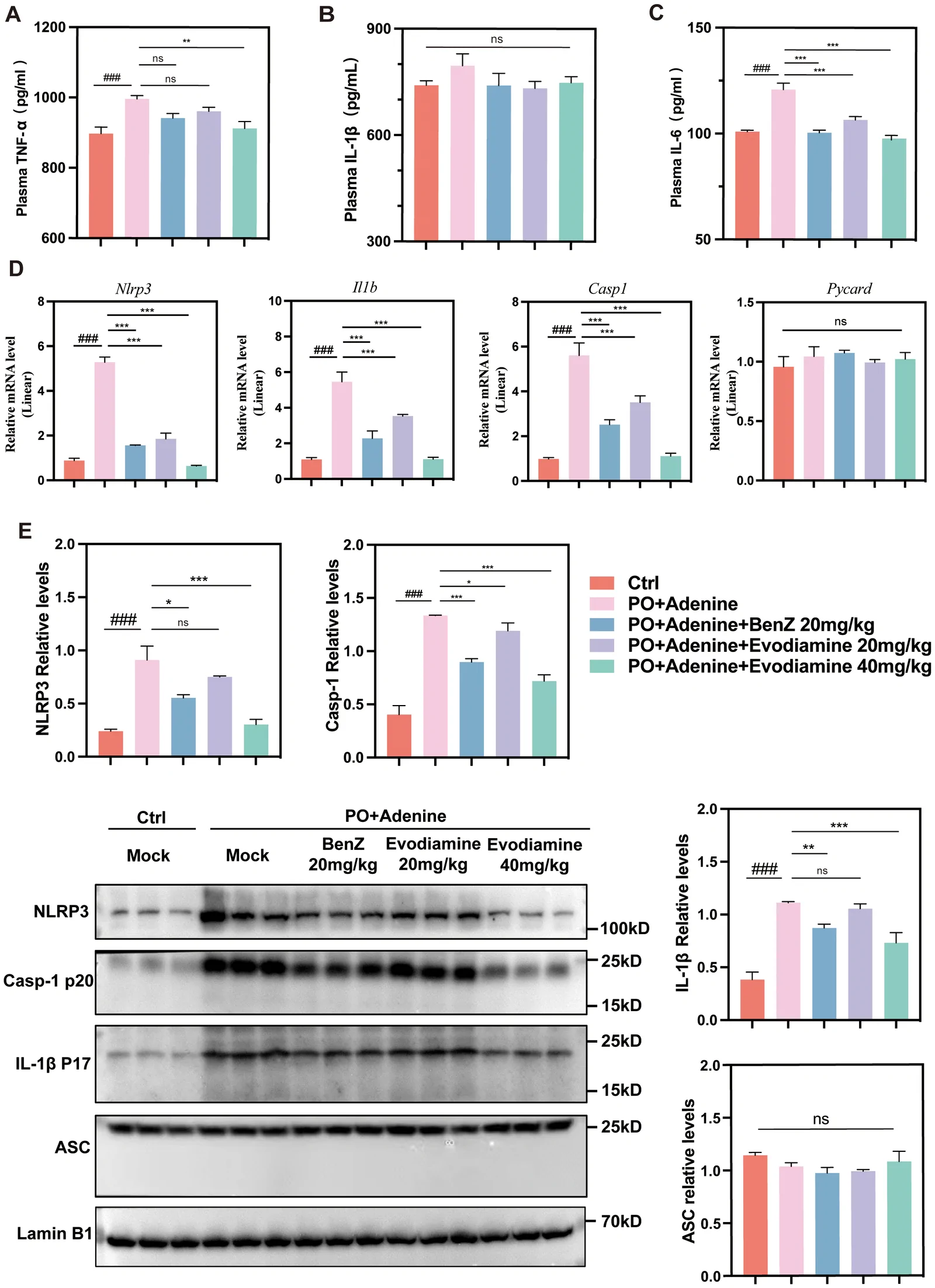
EVO inhibits the activation of NLRP3/Caspas-1/IL-1β. **(A-C)** Plasma TNF-α **(A)**, IL-1β **(B)**, and IL-6 **(C)** production. **(D)** Relative expression of *Nlrp3, Il1b, Casp1*, and *Pycard* mRNA in mice kidney. **(E)** Western blot analysis of NLRP3, Caspase-1, IL-1β, and ASC in mice kidney. Relative intensity of NLRP3, Caspase-1, IL-1β, and ASC were determined. Data are expressed as mean ± SEM from three independent experiments with biological duplicates. ns, not significant. ^###^*P* < 0.001 vs. the group of Ctrl and ^*^P < 0.05, ^**^P < 0.01, ^***^P < 0.001 vs. the group of PO + Adenine.

### EVO alleviates MSU-induced GA and inhibits NLRP3 inflammasome activation

Given that EVO suppressed NLRP3 inflammasome activation in HN mice, we further investigated whether EVO could attenuate MSU-induced acute gouty inflammation, another major pathological manifestation associated with urate deposition. A mouse model of GA was established by intra-articular injection of MSU crystals, and mice were pretreated with EVO at 20 or 40 mg/kg or colchicine at 1 mg/kg for 7 consecutive days before model induction (**Figure 7**). After MSU injection, the model group developed obvious ankle swelling, which appeared at 3 h, peaked at 6 h, and gradually declined thereafter. Compared with the model group, EVO treatment significantly reduced ankle swelling during the observation period, with the 40 mg/kg dose showing a more pronounced inhibitory effect (**Figure 7**). Consistently, gross morphological observation showed marked erythema and edema in MSU-treated mice, whereas EVO pretreatment attenuated these inflammatory manifestations in a dose-dependent manner (**Figure 7**). Histopathological examination further confirmed the protective effect of EVO against MSU-induced joint inflammation. H&E staining showed pronounced synovial hyperplasia, synovial cell damage, and inflammatory cell infiltration in the ankle joints of MSU-treated mice, while these pathological alterations were alleviated by EVO or colchicine pretreatment, particularly in the EVO 40 mg/kg group (**Figure 7C**). Immunohistochemical staining showed increased IL-1β expression in the synovial tissues of MSU-induced mice, which was reduced after EVO treatment (**Figure 7**). In parallel, serum inflammatory and biochemical analyses showed that MSU injection increased the levels of TNF-α, IL-1β, MPO, and UA, whereas EVO, especially at 40 mg/kg, significantly attenuated these changes (**Figure 7E**). To further explore the underlying inflammatory mechanism, NLRP3 inflammasome-related proteins were examined in ankle joint tissues. Western blot analysis revealed that MSU stimulation increased the expression of NLRP3 and cleaved Caspase-1 p20, while EVO treatment dose-dependently suppressed these protein changes (**Figure 7**). Together with the findings in the HN model, these results indicate that EVO exerts protective effects against HUA-related renal and joint inflammation, at least partly through inhibition of NLRP3 inflammasome activation.

**Figure 7:**
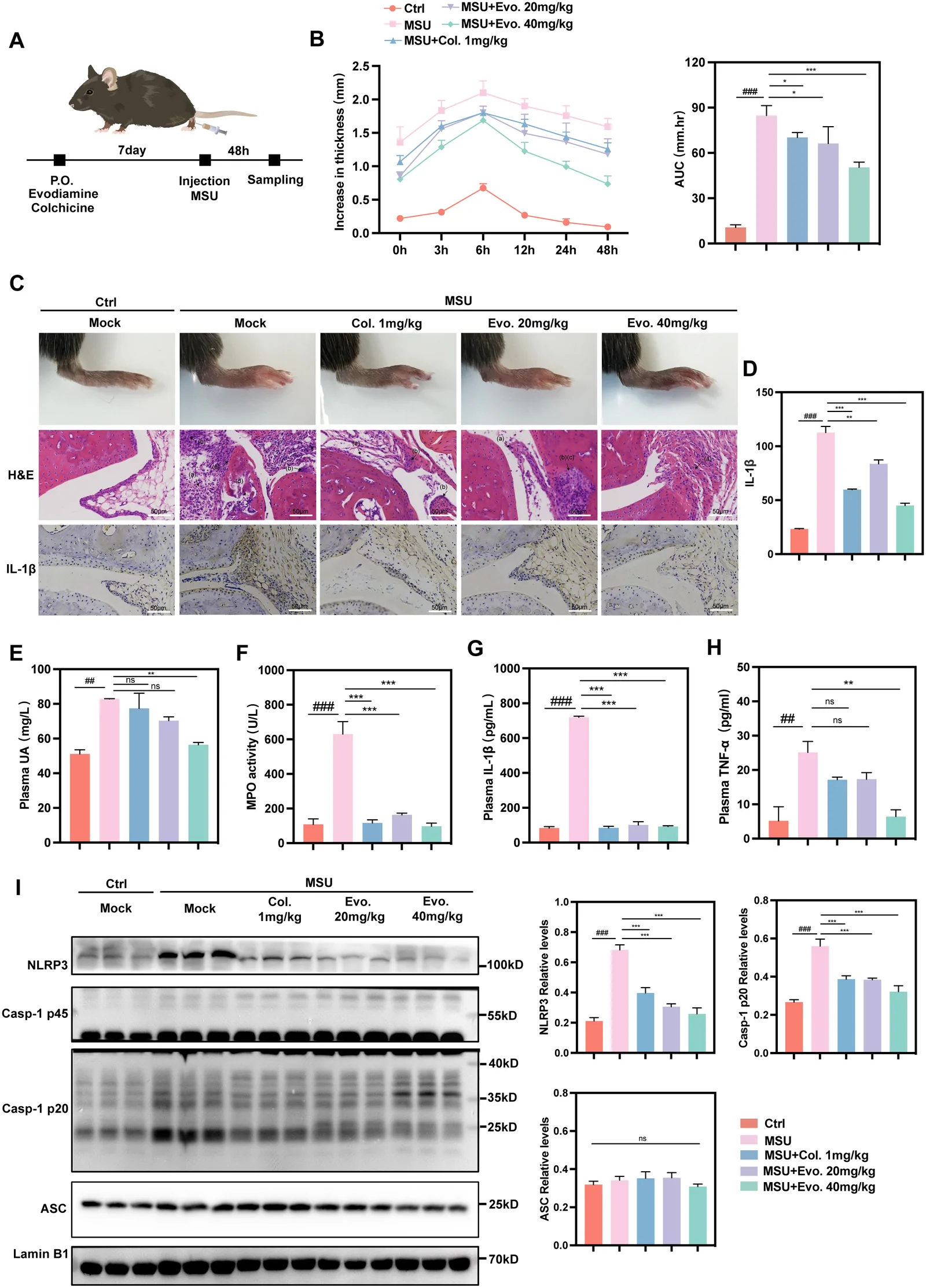
EVO alleviates GA induced by MSU and inhibits the activation of NLRP3 inflammasome. **(A)** Experimental design and treatment schedule. **(B)** Time course of paw thickness increase and area under the curve (AUC) analysis of paw thickness over time. **(C)** Representative images of paw swelling and histological analysis (H&E and IL-1β staining);**(E–H)** Plasma levels of UA **(E)**, MPO activity **(F)**, IL-1β **(G)**, and TNF-α **(H)**; **(I)** Western blot analysis of NLRP3, Caspase-1 p45, Caspase-1 p20 and ASC in paw tissue. Relative intensity of NLRP3, Caspase-1 p20, and ASC were determined. Data are expressed as mean ± SEM from three independent experiments with biological duplicates. ns, not significant. ^##^*P* < 0.01,^###^*P* < 0.001 vs. the group of Ctrl and ^*^P < 0.05, ^**^P < 0.01, ^***^P < 0.001 vs. the group of MSU.

## Discussion

In the present study, we systematically investigated the protective effects of ERWE and the representative pharmacologically active alkaloid EVO in experimental models of HUA, HN, and GA. ERWE reduced circulating UA levels, promoted urinary urate elimination, alleviated hepatic injury, and suppressed systemic inflammatory responses in PO-induced HUA rats. Metabolomic analysis further indicated that ERWE partially restored HUA-associated metabolic disturbances, particularly those related to pyrimidine metabolism, α-linolenic acid metabolism, and butyrate-related pathways. In addition, EVO exerted protective effects in HN and GA models by regulating renal urate transporters, attenuating renal fibrosis, and suppressing NLRP3 inflammasome activation. These findings suggest that ERWE and EVO may protect against HUA-related disorders through coordinated regulation of urate homeostasis, inflammatory responses, and renal pathological remodeling.

ERWE effectively improved UA metabolic disorder in PO-induced HUA rats, as reflected by decreased plasma UA levels and restored urinary UA levels, suggesting that enhanced urinary urate elimination may contribute to its urate-lowering effect. ERWE also reduced AST and ALT levels and decreased circulating inflammatory cells and pro-inflammatory cytokines, indicating that its protective effects were not limited to urate lowering but also involved improvement of HUA-associated hepatic injury and systemic inflammation. Metabolomic analysis showed that ERWE treatment was associated with changes in nucleotide, lipid, energy, and gut microbiota-related metabolic pathways. Pyrimidine metabolism is closely related to nucleotide homeostasis, and changes in orotic acid may reflect broader nucleotide metabolic disturbance under hyperuricemic conditions (31, 32). α-Linolenic acid metabolism may be involved in lipid regulation and inflammatory control, whereas butyrate, an important gut microbial metabolite, has been reported to promote urate excretion partly through ABCG2 regulation in the intestine and kidney (33–35). Therefore, ERWE may improve HUA through integrated regulation of urate elimination and systemic metabolic homeostasis. However, these metabolomic findings remain association-based and require further validation.

EVO showed clear renoprotective effects in PO/adenine-induced HN mice, as evidenced by reduced serum UA and creatinine levels and amelioration of renal pathological injury. EVO reduced renal URAT1 and GLUT9 expression at both the mRNA and protein levels, suggesting that modulation of urate reabsorption may contribute to its urate-lowering effect. Renal *Abcg2* mRNA expression also increased after EVO treatment; however, this finding alone does not establish increased ABCG2 protein abundance or urate efflux. In addition, EVO reduced fibrosis-related markers, including *Col1a1, Acta2, Ctgf, Ldlr*, α-SMA, TIMP1, and LDLR, and attenuated renal collagen deposition, indicating alleviation of renal fibrotic remodeling. The NLRP3 inflammasome is increasingly recognized as an important mediator linking persistent urate overload to renal inflammation and subsequent tissue injury. Urate-associated danger signals can activate the NLRP3 inflammasome, resulting in caspase-1 activation and maturation of IL-1β, thereby amplifying tubulointerstitial inflammation and promoting fibrotic remodeling. A recent study further demonstrated that suppression of NLRP3 inflammasome activation was associated with amelioration of hyperuricemia-induced renal injury (36). Consistent with these findings, EVO reduced renal NLRP3, cleaved Caspase-1 p20, and mature IL-1β p17 expression in the present study, accompanied by attenuation of renal pathological injury and fibrosis-related changes. These findings suggest that suppression of local NLRP3 inflammasome activation may contribute to the renoprotective and anti-fibrotic effects of EVO in HN. However, because specific pharmacological or genetic inhibition of NLRP3 was not performed, the present findings support an association rather than establishing a definitive causal relationship.

The anti-inflammatory effect of EVO was further confirmed in the MSU-induced GA model. MSU crystals are classical activators of the NLRP3 inflammasome and play a central role in acute gouty inflammation. In this study, EVO reduced ankle swelling, synovial hyperplasia, inflammatory cell infiltration, and IL-1β expression in joint tissues. It also decreased serum TNF-α, IL-1β, and MPO levels and inhibited NLRP3 and cleaved Caspase-1 p20 expression in ankle tissues. These results indicate that EVO alleviates acute gouty inflammation mainly by suppressing NLRP3 inflammasome activation and downstream inflammatory responses. Taken together, ERWE improves UA metabolism and systemic metabolic disturbance in HUA rats, whereas EVO alleviates HN and GA by regulating renal urate transporters, inhibiting NLRP3 inflammasome activation, and reducing renal fibrosis. Further studies are required to clarify the direct molecular targets of EVO and validate the causal roles of the identified metabolic pathways.

Several limitations warrant consideration. EVO content in ERWE was not quantified, and the two interventions were not compared in the same HUA model, leaving EVO’s contribution to the extract’s effects unresolved. Differences in species and experimental conditions limit cross-model comparisons, while empirical sample sizes without *a priori* power calculations warrant caution when interpreting non-significant findings. Metabolomics findings remain exploratory because of the small sample size and potential bias from post-treatment sample selection. Further studies should quantify EVO in ERWE, directly compare both interventions, and independently validate metabolic findings using prospectively justified sample sizes and prespecified sampling criteria.

## Conclusion

In conclusion, this study demonstrates that ERWE and its major bioactive constituent EVO exert protective effects against HUA-related disorders *in vivo*. ERWE reduced serum uric acid levels, increased urinary UA concentration, and partially restored metabolic disturbances associated with HUA. EVO further alleviated HN and GA by regulating renal urate transporters, suppressing NLRP3 inflammasome activation, reducing inflammatory responses, and attenuating renal fibrosis. These findings indicate that EVO may represent a potential natural compound for urate-driven renal and inflammatory complications. Further studies are warranted to define its direct molecular targets and evaluate its long-term efficacy and safety.

## Data Availability

The original contributions presented in the study are included in the article/**Supplementary Material**. Further inquiries can be directed to the corresponding authors.

## Supplementary material

Supplementary Figure 1 The base peak chromatogram (BPC) of all the QC samples from plasma **(A)** and Urine **(B)**. X-axis represents the retention time (min), Y-axis represents the intensity of MS (%). ESI (+) and ESI (-) indicate the detections in the positive ion mode and negative ion mode, respectively.

Supplementary Figure 2 PCA and OPLS-DA analysis of urinary and plasma metabolites across different treatment groups in ESI (+). PCA scores plot of urinary **(A)** and plasma **(B)** metabolites between Ctrl, Model and ERWE group; OPLS-DA scores plot of urinary **(C)** and plasma **(D)** metabolites between Ctrl, Model and ERWE group; OPLS-DA analysis of urinary **(E)** and plasma **(F)** metabolites comparing Ctrl vs. Model and Model vs. ERWE.

Supplementary Figure 3 KEGG pathway topology analysis of urine and plasma metabolites in HUA rats and ERWE intervention. **(A, B)** KEGG topology analysis of urinary **(A)** and plasma **(B)** metabolites in HUA rats with ERWE intervention.
