# Supplementary material for: Evodiamine attenuates hyperuricemia-associated gouty arthritis and renal injury through urate-inflammatory axis modulation
Source: Front Immunol. 2026 Oct 22;17:1932250. doi: 10.3389/fimmu.2026.1932250 (PMC13735098; doi:10.3389/fimmu.2026.1932250)
Supplement: Supplementary file 1 [file SupplementaryFile1.pdf]

Evodiamine attenuates hyperuricemia-associated gouty arthritis and renal injury through urate-inflammatory axis modulation

Yu Mou[1,*], Wenhao Liao[2,*], Qun Niu[1*], Xue Zhan[1], Tianxin Zhang[3], YuChen Li[1,4], Hong Che[1,5], Peijia Liu[1], Xiangdong Yang[1,5,#], Jianyuan Tang[1,6,#], Zhilei Wang[1,7,#]

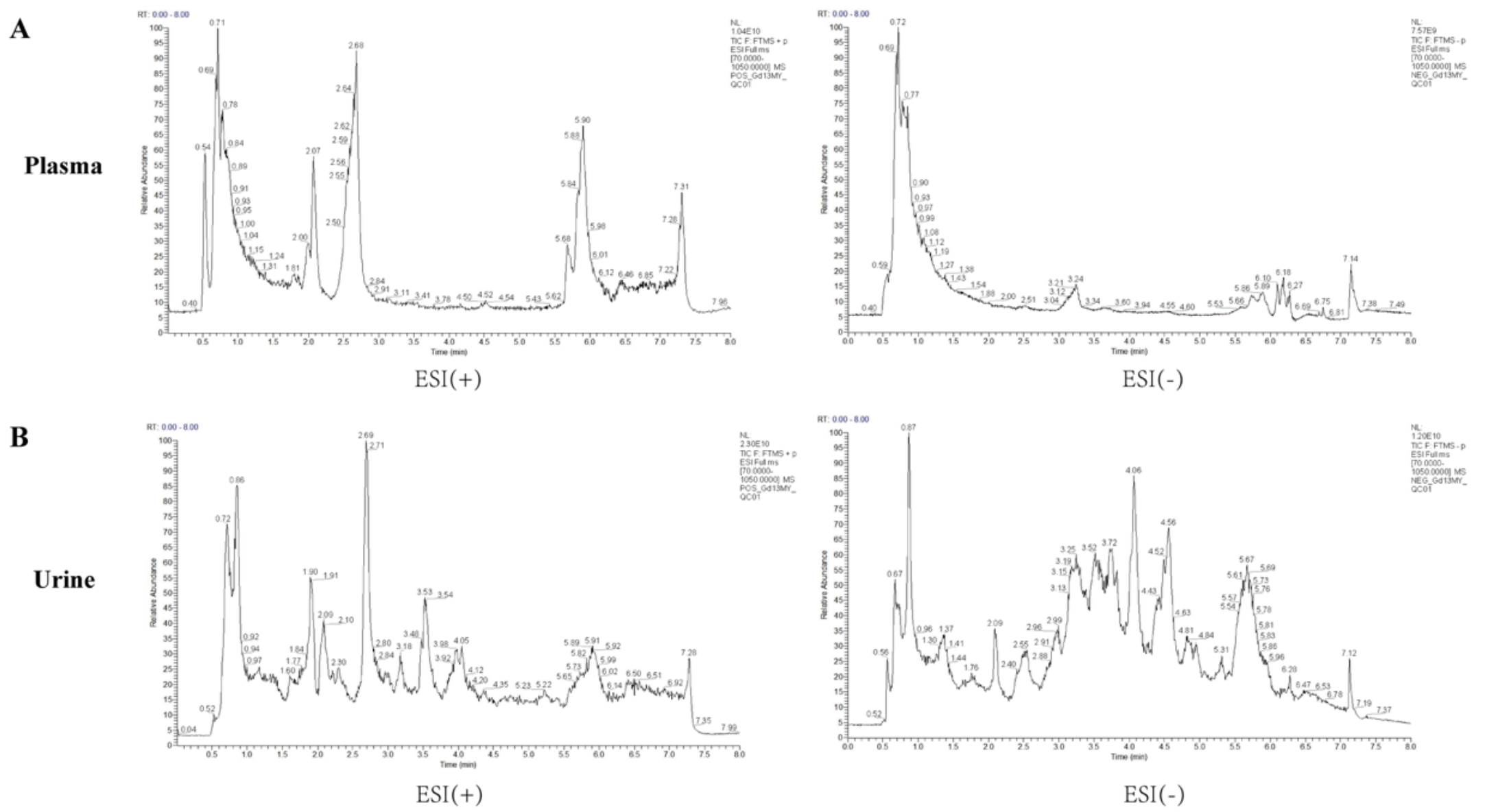

Figure S1 The base peak chromatogram (BPC) of all the QC samples from plasma (A) and Urine (B). X-axis represents the retention time (min), Y-axis represents the intensity of MS (%). ESI (+) and ESI (-) indicate the detections in the positive ion mode and negative ion mode, respectively.

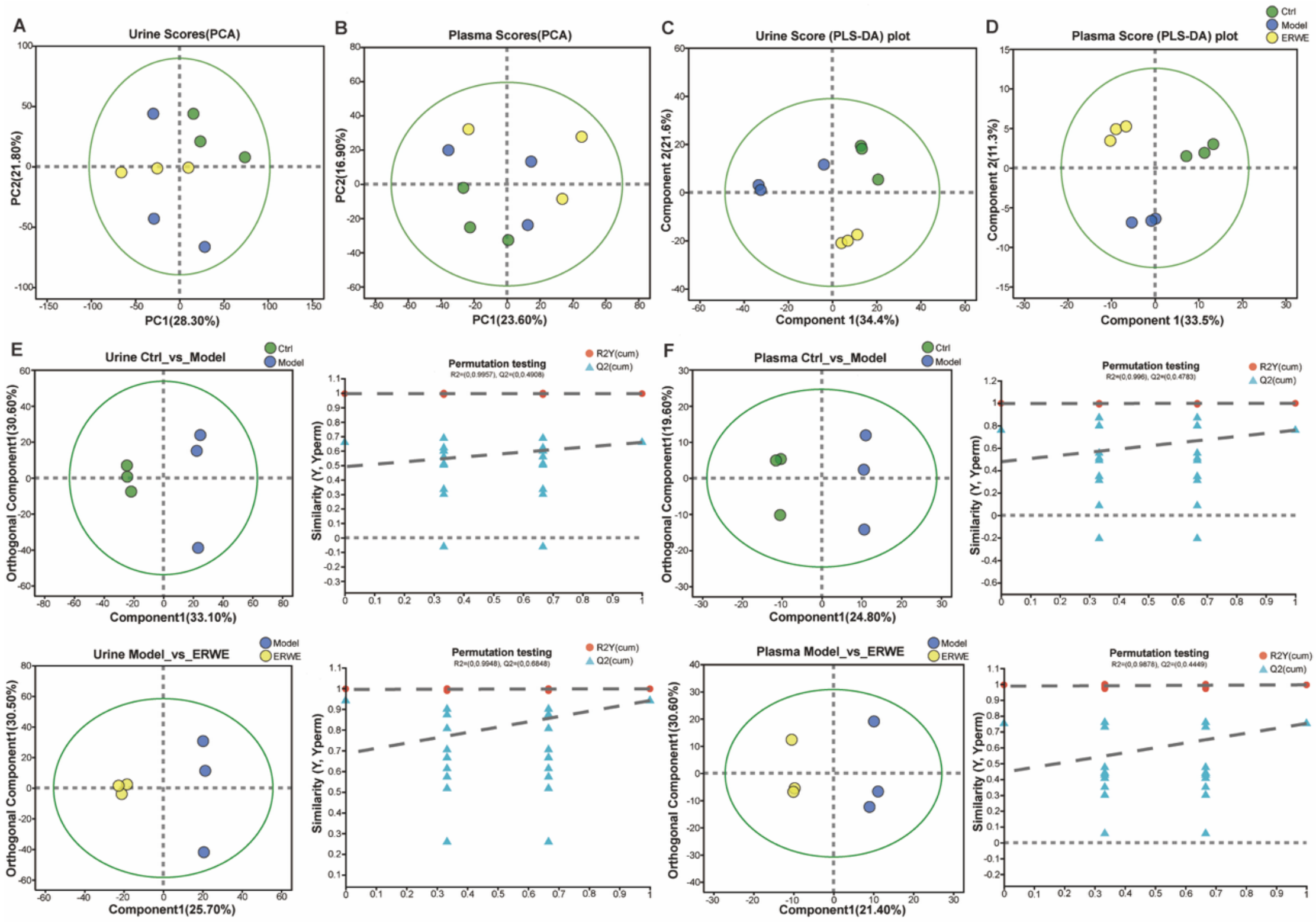

Fig.S2 PCA and OPLS-DA analysis of urinary and plasma metabolites across different treatment groups in ESI (+). PCA scores plot of urinary (A) and plasma (B) metabolites between Ctrl, Model and ERWE group; OPLS-DA scores plot of urinary (C) and plasma (D) metabolites between Ctrl, Model and ERWE group; OPLS-DA analysis of urinary (E) and plasma (F) metabolites comparing Ctrl vs. Model and Model vs. ERWE.

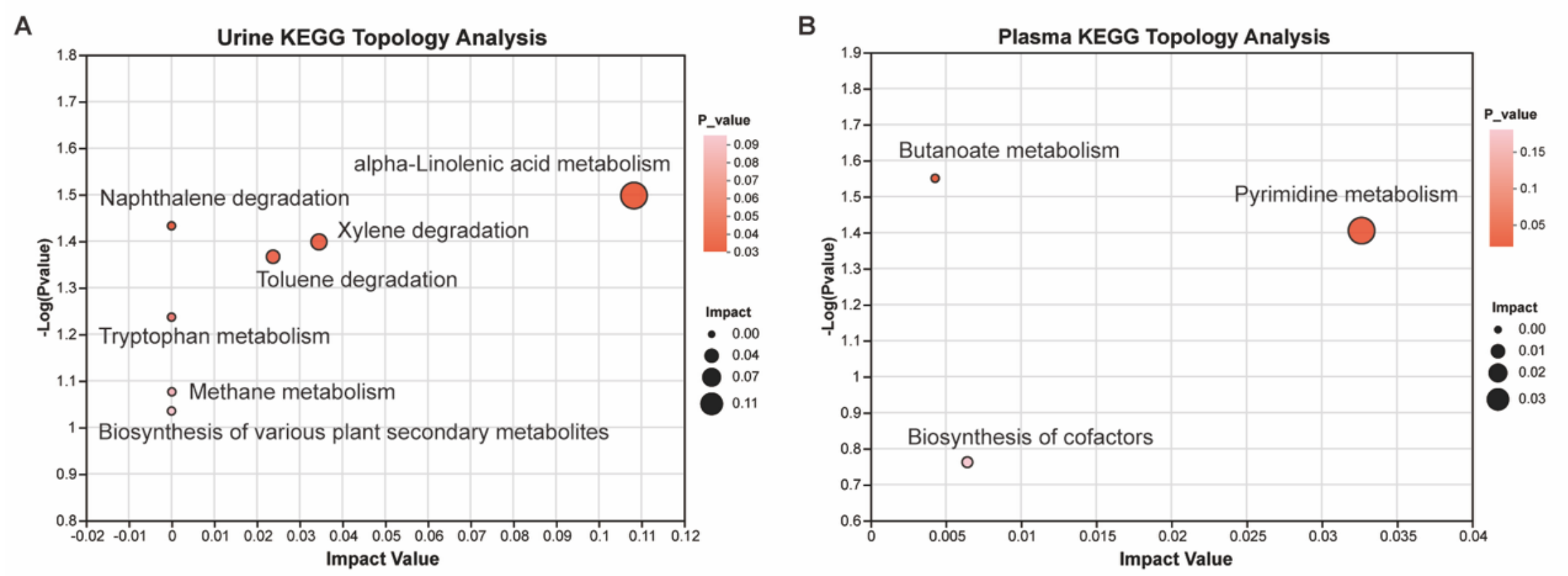

Fig.S3. KEGG pathway topology analysis of urine and plasma metabolites in HUA

rats and ERWE intervention. (A, B) KEGG topology analysis of urinary (A) and plasma (B) metabolites in HUA rats with ERWE intervention.
